# Dynamic relationships between psychological capital and adaptation in new military recruits: a longitudinal cross-lagged panel network analysis

**DOI:** 10.3389/fpsyt.2025.1691043

**Published:** 2025-10-23

**Authors:** Qiao Zhang, Chen Xu, Min Li

**Affiliations:** Department of Military Psychology, Faculty of Medical Psychology, Army Medical University (Third Military Medical University), Chongqing, China

**Keywords:** psychological capital, adaptation, new recruits, military training, cross-lagged panel network

## Abstract

New recruits face severe psychological problems due to military adaption. This study examined the dynamic interplay between psychological capital (PsyCap) and adaptation among recruits during their initial training period. A total of 988 male recruits were assessed at three time points over an 8-week period, with cross-lagged panel network (CLPN) analysis employed to model predictive pathways and identify key nodes. Optimism had the strongest impact on the early adaptation (T1→T2), acting as a central linking bridge between PsyCap and the adaptation dimensions. Later in the T2→T3 stage, hope emerged as the strongest predictor, especially in relation to the reduction of the physical and psychological symptoms and an improvement in the role cognition. Resilience was a robust predictor of symptom improvement. The results reveal evolving roles of PsyCap components during adaptation, highlighting the importance of targeted interventions for enhancing adaptation among new recruits.

## 1 Introduction

Adapting to a new environment is an essential milestone in the growth and development of an individual. The highly structured, disciplined and high-pressure nature of the military environment makes it more difficult to adapt. When recruits are enlisted, their lifestyle changes dramatically and they may lose their social support system for a while. In the closed barracks, they must quickly get used to strict schedules, intensive physical training, and behavioral regulations, all of which are closely linked to elevated anxiety levels and adaptation difficulties (1, 2). Researches showed that, according to the Chinese military, the early stages of training are associated with a high risk of developing psychological assimilation problems. Some people may have significant psychological stress reactions and meet the criteria for clinical diagnosis of adaptation disorders (1, 3). According to a longitudinal study involving the basic training of the US Army infantry soldiers, their anxiety levels were observed to dynamically change as the training time progressed (4). Health monitoring data from the US military indicate that the first six months after enlistment represent a period of elevated incidence of adjustment disorders (5). Approximately 60% of soldiers diagnosed with an adjustment disorder early in their career will experience an early end to their military career within the next two years, and 24.3% will be diagnosed with other mental disorders at follow-up (5, 6). All in all, findings reveal the first stage of military service is a particularly vulnerable stage for adaptation. It emphasizes the need to detect and strengthen protective psychological resources, which is a prerequisite for the effective design of adjustment-promoting interventions for recruits.

Adaptation can be understood as a multidimensional construct encompassing role identity, environmental adjustment, physical and psychological health and behavioral regulation, shaped by the dynamic interaction between individuals and their surrounding context (7–9). The adaptation of recruits is not only influenced by external factors such as environmental pressure and social support, but also depends on the internal psychological resources. From the perspective of traditional psychology, management for adaptation issues have mainly revolved around symptom reduction. However, dual continuum model indicates that positive and negative affect are not two ends of a single bipolar continuum but rather represent two independent constructs, low levels of symptom do not mean high well-being (10, 11). Hence, simply eliminating problems is not sufficient to achieve good adaptation and enhancing positive psychological resources is also necessary. Positive psychology advocates the development of an individual’s strengths and positive capabilities; it supports the strengthening of positive psychological resources in organizational and training contexts (12). In the context of positive psychology, psychological capital (PsyCap) is regarded as an important and malleable resource, consisting of four core elements: efficacy (having confidence to take on and put in the necessary effort to succeed at challenging tasks), optimism (being a generalized positive expectancy and an attributional style that integrates past, present, and future perspectives), hope (persevering toward goals and, when necessary, redirecting pathways to reach them), and resilience (when beset by problems and adversity, sustaining and bouncing back to attain success) (13, 14). Finch et al. have found that optimism and efficacy increase significantly following short interventions, whereas hope and resilience exhibit slower growth (15). Hope reflects longer-term goal pursuit and pathway thinking, including agency and planning components distinct from optimism (16). Resilience functions as a relatively stable buffer that mitigates distress across phases of training (17). Studies in multiple contexts consistently show that higher levels of PsyCap can lead to increased positive adaptability in terms of attitude, behavior and performance, as well as maintain a more stable level of performance and adaptability (18, 19). In military populations, PsyCap correlates with indicators such as organizational identification, job satisfaction, and retention stability. This relationship has been confirmed numerous times in the sample of Chinese military personnel (20, 21). Particularly in special environments such as plateau stationing, the PsyCap of individuals has been reported to exert a buffering effect on psychological-behavioral performance under stress (22).

Due to its characteristic of being state-dependent, PsyCap can be effectively enhanced through short-term interventions. PsyCap intervention not only improves the level of PsyCap, but also enhances subjective well-being and reduces negative emotions (23, 24). In our previous study with military personnel, we observed that PsyCap training resulted in short-term enhancements in positive psychological resources and mental health which can easily be scaled up and low-cost (25). Although cross-sectional studies have shown that PsyCap is associated with adaptation, traditional variable-centered analysis cannot reveal the details of their interaction, the direction of the relationship, and their dynamic changes at different stages (26). In contrast, cross-lagged panel network (CLPN) not only reflects the correlation structure of variables across different time points but also highlights the directionality of predictive relationships, the temporal dynamics of change, and the identification of bridge variables that serve as key drivers in the system (27). These features make CLPN uniquely suited to uncovering how PsyCap dimensions interact with adaptation over time in a training environment. Therefore, this study employs CLPN to investigate the dynamic interplay between recruits’ PsyCap and adaptation during training, aiming to provide scientific guidance for phased and targeted interventions.

## 2 Participants and methods

### 2.1 Participants

**Figure 1:**
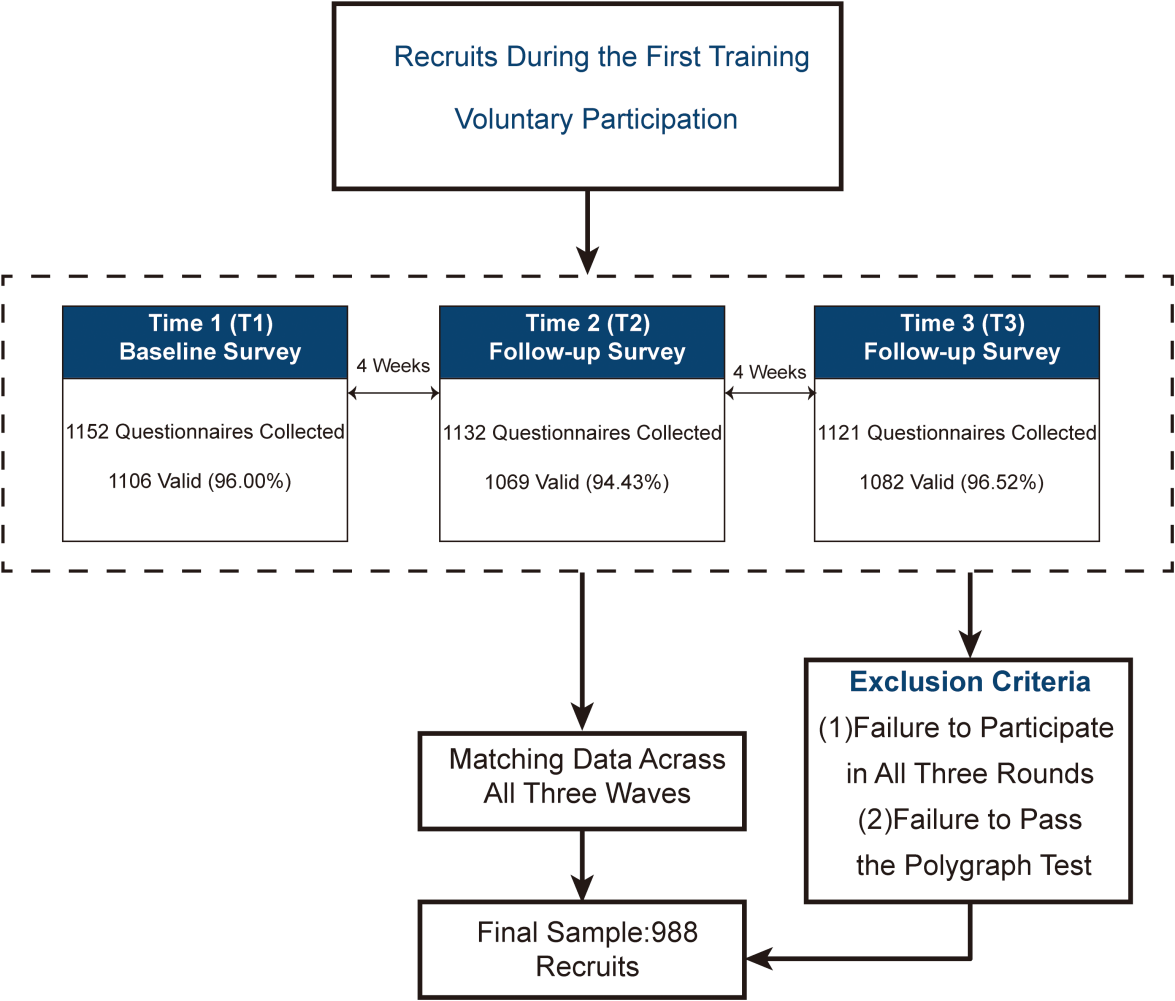
Flowchart of recruit longitudinal follow-up survey design.

A cluster sampling method was employed, with two companies of newly enlisted recruits from a training base selected as the study sample. A three-wave longitudinal follow-up survey was conducted at 4-week intervals over an 8-week period. The inclusion criteria of the participants were: 1) recruits during the first training period; 2) voluntary participation. Exclusion Criteria were: 1) failure to participate in all three rounds of data collection; 2) failure to pass the polygraph test. At Time 1 (T1), 1,152 questionnaires were collected, of which 1,106 were valid (response rate: 96.00%); at Time 2 (T2), 1,132 were collected, with 1,069 valid responses (response rate: 94.43%); and at Time 3 (T3), 1,121 were collected, with 1,082 valid responses (response rate: 96.52%). After matching data across all three waves, a final sample of 988 recruits (all male) was included in the analysis, detailed baseline data for all samples are provided in **Supplementary Table S1**. A detailed flowchart of the study could be found in **Figure 1**. The study was approved by the institutional ethics committee (Approval No. 2020-019-02), and informed consent was obtained from all participants.

### 2.2 Measuring tools

#### 2.2.1 Recruit adaptation questionnaire

The recruit adaptation questionnaire developed by Wang et al. (28) was used to assess recruits’ adaptation. This instrument consists of 36 items covering five dimensions: military role perception, interpersonal relationships, physical and psychological symptoms, military environment adaptation, coping strategies. Items are rated on a 5-point Likert scale (1 = completely inconsistent to 5 = completely consistent), with higher scores indicating poorer adaptation. The questionnaire has demonstrated good reliability and validity among new recruit in China (29). In our study, the Cronbach’s α coefficient for the total scale was 0.924.

#### 2.2.2 Positive Psychological Capital Questionnaire

PsyCap was assessed using the 26-item Positive Psychological Capital Questionnaire (PPQ) developed by Zhang et al. (30), which was adapted from the original PPQ proposed by Luthans et al. (13). This 26-item instrument comprises four dimensions: hope (e.g., I work actively to realize my ideals), optimism (e.g., When situations are uncertain, I always expect the best), efficacy (e.g., I am confident in my abilities), and resilience (e.g., I can recover quickly from setbacks). Items are rated on a 7-point Likert scale (1 = completely inconsistent to 7 = completely consistent), with higher scores indicating higher levels of psychological capital. The PPQ has demonstrated good reliability and validity among military personnel (31). In our study, the Cronbach’s α coefficient for the total scale was 0.929.

### 2.3 Statistical analysis

#### 2.3.1 Descriptive analysis

Descriptive statistics were performed using SPSS version 26.0. Means and standard deviations of adaptation and PsyCap dimension scores were calculated, and repeated-measures analyses of variance were performed to preliminarily examine changes across the three time points.

#### 2.3.2 Network estimation

Network modeling and visualization were conducted in R version 4.4.2, following established recommendations for CLPN analysis (32). Cross-lagged panel networks for T1→T2 and T2→T3 were constructed using the ‘*glmnet’* package (33). LASSO (Least Absolute Shrinkage and Selection Operator) regression was applied for regularization, with alpha set to 1 to impose the L1 penalty. For each prediction model, the optimal λ value (lambda.min), which minimized cross-validation error, was selected. All predictor variables were standardized to ensure comparability of the LASSO penalty (33). In the network plots, nodes represented dimensions, while edges indicated associations between them. Edge thickness reflected connection strength, with blue and red edges denoting positive and negative associations, respectively. Directed arrows indicated temporal relationships, and auto-regressive effects were constrained to zero to isolate cross-lagged effects. To facilitate visual comparison of networks, we applied the averageLayout function from the *‘qgraph’* package (34).

#### 2.3.3 Network inference

Node centrality was quantified using out-expected influence (out-EI), in-expected influence(in-EI), and bridge-expected influence(bridge-EI): out-EI indexes the extent to which a node predicts other nodes at the next wave; in-EI indexes the extent to which a node is predicted by other nodes; and bridge-EI indexes the predictive influence a node exerts on nodes in other communities (35–37).

#### 2.3.4 Network accuracy and stability

To evaluate stability, we used the *‘bootnet’* package to compute the correlation stability (CS) coefficient. CS ≥ 0.50 indicates good stability, CS between 0.25-0.50 suggests at least adequate stability of centrality estimates, and CS < 0.25 indicates instability (38). Edge weight accuracy was tested through nonparametric bootstraps (1000 samples; 95% confidence intervals), with narrower intervals showing higher accuracy (38). Bootstrap difference tests (1000 samples, *α* = 0.05) were conducted to compare edge weights and centrality indices (38).

## 3 Result

### 3.1 Descriptive statistics

Abbreviations, descriptive statistics, and results of the difference tests for each dimension of psychological capital and adaptation are presented in **Table 1**. Repeated-measures ANOVA revealed that certain nodes in the network increased significantly over time, including efficacy (*F* = 92.116, *p* < 0.001) and optimism (*F* = 45.697, *p* < 0.001), whereas hope showed no significant temporal change. Different aspects of adaptation exhibited distinct temporal patterns: scores for military role perception (*F* = 9.278, *p* < 0.001), physical and psychological symptoms (*F* = 141.612, *p* < 0.001), and military environment adaptation (*F* = 21.167, *p* < 0.001) improved over time (i.e., lower scores indicated better adaptation), while interpersonal relationships and coping strategies did not show significant changes.

**Table 1:** Node labels and descriptive statistics at different time points.

Item	Label	T1		T2		T3		Repeated measures ANOVA		
		*M*	*SD*	*M*	*SD*	*M*	*SD*	*F*	*p*	Bonferroni
Efficacy	PC1	5.33	0.87	5.45	0.95	5.61	0.89	92.116	<0.001	T1<T2<T3
Resilience	PC2	5.46	1.00	5.45	1.11	5.59	1.03	20.331	<0.001	T1=T2<T3
Optimism	PC3	5.77	0.82	5.91	0.88	5.96	0.83	45.697	<0.001	T1<T2<T3
Hope	PC4	6.17	0.78	6.18	0.80	6.20	0.77	1.217	0.296	/
Military Role Perception	A1	11.45	3.50	11.74	3.90	11.34	3.72	9.278	<0.001	T1=T3<T2
Interpersonal Relationships	A2	7.60	2.34	7.59	2.55	7.47	2.48	2.372	0.097	/
Physical and Psychological Symptoms	A3	15.60	5.96	16.37	6.57	13.76	5.65	141.612	<0.001	T3<T1<T2
Military Environment Adaptation	A4	18.76	5.54	18.40	5.88	17.89	5.86	21.167	<0.001	T3<T2<T1
Coping Strategies	A5	12.20	3.18	11.98	3.38	12.05	3.35	3.001	0.051	/

PC, Psychological Capital; A, Adaptation; M, mean; SD, standard deviation. *p* values are based on repeated measures ANOVA; Bonferroni tests indicate pairwise comparisons among T1, T2, and T3.

### 3.2 Cross-lagged panel network analysis

#### 3.2.1 Network estimation

**Figure 2:**
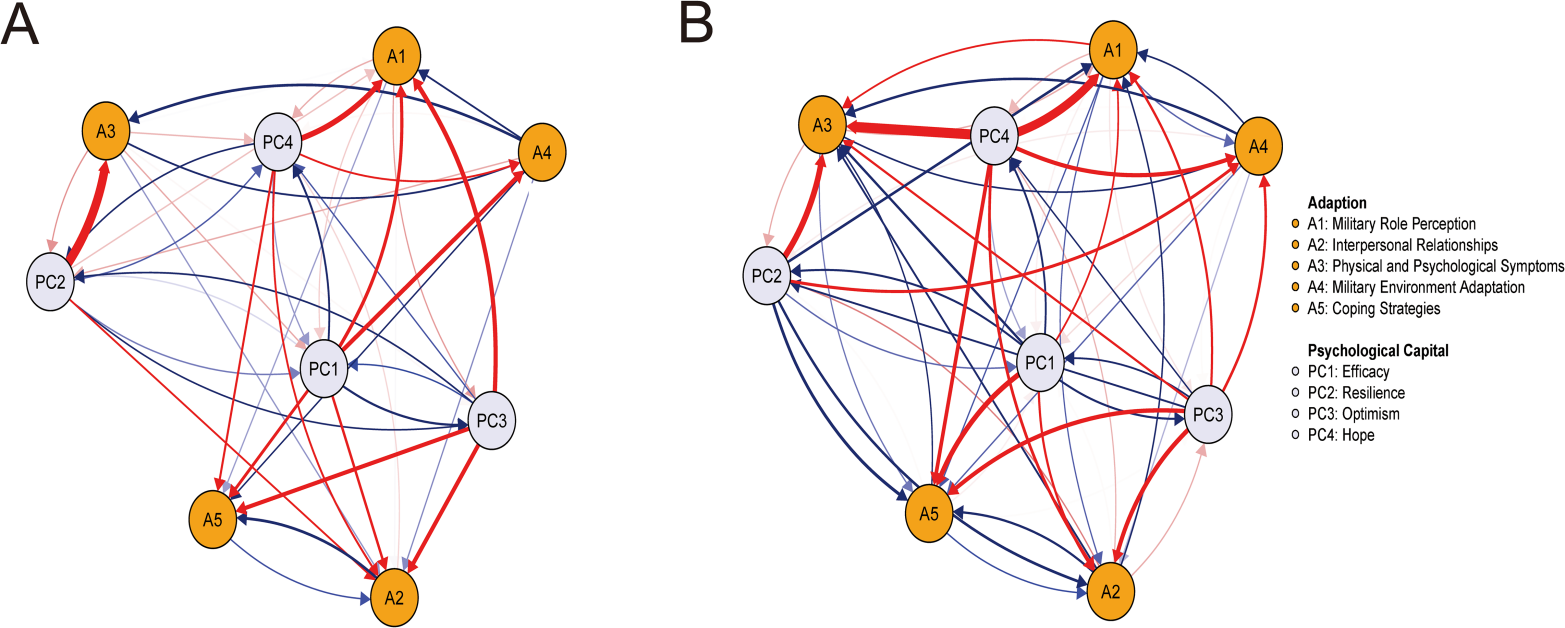
The cross-lagged panel networks for T1→T2 and T2→T3. **(A)** T1→T2; **(B)** T2→T3. PC1 = efficacy, PC2 = resilience, PC3 = optimism, PC4 = hope; A1 = military role perception, A2 = interpersonal relationships, A3 = physical and psychological symptoms, A4 = military environment adaptation, A5 = coping strategies. Arrows demonstrate unique longitudinal relationships. Blue edges indicate positive relationships, and red edges indicate negative relationships. Edge thickness reflects connection strength. And auto-regressive effects were constrained to zero to isolate cross-lagged effects.

**Figure 2** presented the cross-lagged networks from T1→T2 and T2→T3 after removing autoregressive effects (the networks retaining autoregressive effects were shown in **Supplementary Figure S1**). As shown in **Figure 2A**, The T1→T2 network comprised 55 edges (31 positive, 24 negative). Longitudinally, resilience exerted the strongest negative predictive effect on physical and psychological symptoms (PC2→A3, β = –0.780). It indicated higher resilience at T1 demonstrates the strongest association with fewer physical and psychological symptoms (lower A3) at T2. Optimism strongly predicted military role perception (PC3→A1, β = –0.360), interpersonal relationships (PC3→A2, β = –0.320), and coping strategies (PC3→A5, β = –0.280). Given that higher adaption scores denoted poorer adaptation, the negative β implied that higher optimism at T1 significantly predicted better adaptation in military role perception, interpersonal relationships and coping strategies (lower A1, A2, A5) at T2. Hope showed a strong negative predictive effect on military role perception (PC4→A1, β = –0.410), while efficacy significantly predicted military environment adaptation (PC1→A4, β = –0.310) and military role perception (PC1→A1, β = –0.250), suggesting that higher levels of hope at T1 were linked to better military role perception (lower A1), while greater efficacy at T1 demonstrated a positive longitudinal association with adaptation in military environment and military role perception (lower A4, A1). The T2→T3 network (**Figure 2B**) included 63 edges (35 positive, 28 negative). Hope exerted the strongest negative predictive effects on physical and psychological symptoms (PC4→A3, β = –0.970) and military role perception (PC4→A1, β = –0.850), suggesting that higher levels of hope at T2 demonstrated the strongest association better adaptation in physical and psychological symptoms and military role perception (lower A3 and A1) at T3. Resilience also strongly predicted physical and psychological symptoms (PC2→A3, β = –0.610), indicating higher resilience at T2, fewer physical and psychological symptoms at T3 (lower A3). Efficacy most strongly predicted coping strategies (PC1→A5, β = –0.470), while optimism strongly predicted interpersonal relationships (PC3→A2, β = –0.410). suggesting that higher efficacy at T2 predicted better adaption in coping strategies (lower A5) at T3, while higher optimism at T2 predicted better adaption in interpersonal relationships (lower A2) at T3.

#### 3.2.2 Centrality analysis

**Figure 3:**
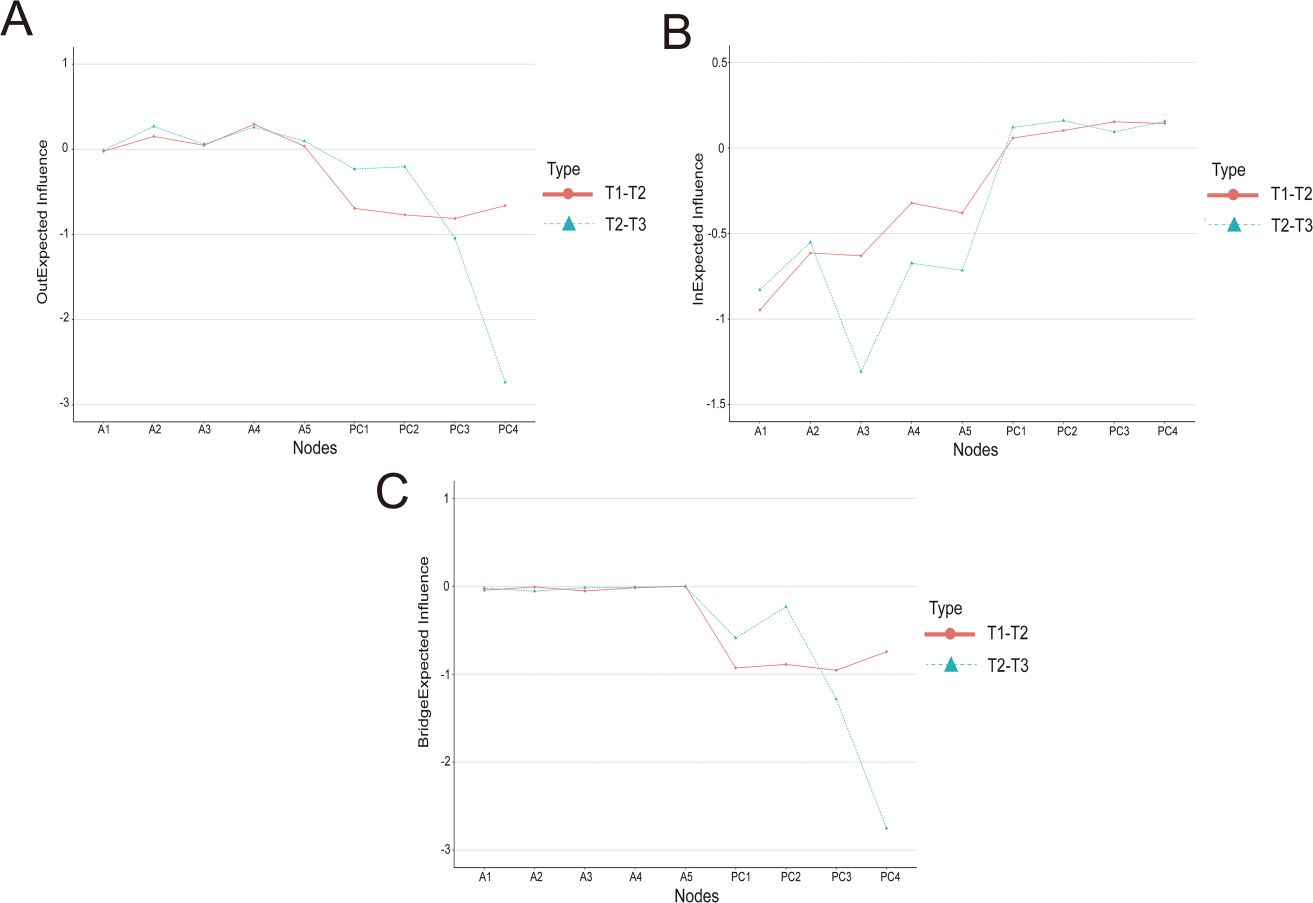
Out-expected influence, in-expected influence and bridge expected influence in the CLPN. **(A)** Out-expected influence; **(B)** In-expected influence; **(C)** Bridge-expected influence. PC1 = efficacy, PC2 = resilience, PC3 = optimism, PC4 = hope; A1 = military role perception, A2 = interpersonal relationships, A3 = physical and psychological symptoms, A4 = military environment adaptation, A5 = coping strategies.

The centrality indices of the cross-lagged network models were shown in **Figure 3** (A: out-EI; B: in-EI; C: bridge-EI). In the T1→T2 network, optimism (PC3) had the highest out-EI, followed by resilience (PC2), efficacy (PC1), and hope (PC4). Within adaptation, military role perception (A1) had the highest in-EI, followed by physical and psychological symptoms (A3) and interpersonal relationships (A2). Optimism (PC3) showed the highest bridge-EI. In the T2→T3 network, hope (PC4) and optimism (PC3) both exhibited the highest out-EI. Physical and psychological symptoms (A3) had the highest in-EI, followed by military role perception (A1). Hope (PC4) also had the highest bridge-EI.

#### 3.2.3 Stability and difference testing

Case-drop bootstrap results indicated good stability of the centrality indices in both cross-lagged networks (**Supplementary Figures S2**, **S3**). Specifically, the CS coefficient for in-EI was 0.516 (T1→T2) and 0.672 (T2→T3); for out-EI, 0.283 (T1→T2) and 0.516 (T2→T3); and for bridge-EI, 0.283 (T1→T2) and 0.516 (T2→T3). All values exceeded the 0.25 threshold, demonstrating that the estimated centrality indices were stable. Bootstrapped 95% confidence intervals for edges were provided in **Supplementary Figures S4**, **S5**; the narrow intervals indicated that the edge-weight estimates were precise. Difference tests (**Supplementary Figures S6**–**S13**) further revealed that during T1→T2, the out-EI scores of all four PsyCap dimensions were significantly higher than those of most adaptation dimensions, while no significant differences were found among the four PsyCap components themselves. During T2→T3, both hope and optimism showed significantly higher out-EI and bridge-EI than all other nodes.

## 4 Discussion

We utilized the CLPN approach to examine the dynamic interplay between psychological capital and adaptation throughout the initial training period of recruits. The results identified distinct temporal patterns in the core components of PsyCap: efficacy and optimism increased significantly over time, whereas hope remained relatively stable. In terms of adaptation, progressive improvements were observed in military role perception, physical and mental symptoms, and military environment adaptation, while no significant changes emerged in interpersonal relationships or coping strategies. Analysis revealed that optimism emerged as a central bridging node between PsyCap and adaptation during the early training stage. In the later stage, both hope and optimism jointly occupied key positions within the network. Notably, resilience consistently predicted reductions in physical and mental symptoms across both time points. Together, these findings expand longitudinal evidence linking PsyCap to adaptation in high-stress training contexts and support the design of phased and targeted psychological interventions in military settings.

The longitudinal changes observed in components of PsyCap revealed significant increases in efficacy and optimism, consistent with previous studies. Notably, empirical researches on college freshmen and military recruits have demonstrated that environment characterized by clear goals, moderate difficulty, and timely feedback can effectively foster improvements in these two components through the accumulation of mastery experiences and external validation (17, 23, 39, 40). This process is consistent with Bandura’s social cognitive theory, which highlights mastery experiences and social persuasion as key pathways in shaping efficacy beliefs. Clear achievement cues and positive reinforcement strengthen the internalized sense of “I can do it,” thereby promoting more adaptive attributions and future-oriented expectations (41). Within the 8-week intensive training context of the present study, strict military routines and rigorous assessment systems offered recruits clear performance standards and frequent feedback on success, making the concurrent improvement in efficacy and optimism both theoretically and empirically expected. In contrast, hope relies more on mid- to long-term goal setting, pathway planning, and flexible thinking. In a PsyCap training study, Justin et al. reported that hope exhibited only modest improvement over a six-week follow-up period, in contrast to the immediate gains observed in efficacy and optimism (42). Similarly, a recent meta-analysis of 41 controlled trials reported that hope consistently yielded the smallest Cohen’s d among the four PsyCap dimensions (43). Taken together, the limited change in hope observed here may therefore reflect the longer developmental trajectory required for this dimension.

As training progressed, significant improvements were observed in military role perception, physical and mental symptoms, and military environment adaptation, suggesting that recruits gradually internalized their military identity and demonstrated adaptive gains in both physical capacity and psychological resilience (44). These improvements may reflect the influence of stable personality traits and prior socialization experiences, which are less likely to shift over a short period of time (45–47). Moreover, the relatively limited scope of interpersonal interactions in the military setting, combined with the structured nature of peer and hierarchical relationships, may further constrain the short-term malleability of these aspects. To enhance interpersonal adjustment and coping strategies during the initial training phase, psychological intervention programs may benefit from incorporating dedicated modules such as conflict management and emotional regulation training (48, 49).

Our network analysis further elucidated the functional pathways of PsyCap components in the adaptation process of recruits, highlighting a progression from key nodes to bridging variables and ultimately to adaptive outcomes. During the T1 to T2 phase, optimism showed the highest bridge centrality, suggesting that the generalized positive expectancy may serve as a key psychological mechanism linking to subsequent adaptation outcomes. This finding aligns with meta-analytic evidence from Avey et al., which identified optimism as a central element in the relationship between PsyCap and desirable outcomes (18). In the T2 to T3 phase, both hope and optimism emerged as core nodes, suggesting that after initial adjustment, their combined influence becomes crucial for maintaining adaptive functioning. This observation echoes prior longitudinal research, which found that hope and optimism jointly predict reductions in anxiety and improvements in adjustment (50). Meanwhile, resilience consistently predicted reductions in physical and mental symptoms across both phases, underscoring its role as a stable psychological protective factor. This finding aligns with evidence from longitudinal studies and structural equation modeling in various military samples, which have shown that resilience reliably predicts lower stress, depression, and burnout over time (17, 51).

The findings of this study carry important practical implications. Given the state-like nature of psychological capital, targeted psychological interventions can be implemented at different stages of military training. In the early phase, efforts should focus on enhancing optimism and resilience to facilitate role adjustment and alleviate physical and psychological distress. In the later stages, strengthening hope and optimism may help sustain motivation and adaptive functioning. Such short-term intervention is a viable candidate for integration into routine training programs, enabling a more precise enhancement of adaptation. Several limitations of this study should be acknowledged. First, the sample consisted solely of male recruits from a single basic training site, which limits the generalizability of the findings to female recruits and other branches of the military. Second, although the CLPN reveals predictive and directional associations, causal inferences remain tentative and require confirmation through experimental or intervention-based designs. Third, potential confounders such as age, or place of origin were not adjusted for in the CLPN analysis because of the sample’s relative homogeneity and to avoid overcomplicating the model. Future work should incorporate these variables to more precisely estimate the unique contributions of PsyCap to adaptation. Finally, the follow-up period covered only the first two months of enlistment. Extending the observation window into the later stages of service would provide deeper insight into the long-term stability and impact of the PsyCap–adaptation dynamic.

## 5 Conclusion

This study revealed the dynamic interplay between PsyCap and adaptation in new recruits, highlighting the stage-specific predictive roles of PsyCap components. Optimism and hope emerged as key resources at different stages, while resilience consistently functioned as a stable protective factor.

## Data Availability

The datasets presented in this article are not readily available because the datasets generated and/or analyzed during the current study are not publicly available due to confidentiality restrictions but are available from the corresponding author in a de-identified form upon reasonable request. Requests to access the datasets should be directed to Min Li, limin52267@tmmu.edu.cn.
