# Supplementary material for: Dynamic relationships between psychological capital and adaptation in new military recruits: a longitudinal cross-lagged panel network analysis
Source: Front Psychiatry. 2025 Oct 23;16:1691043. doi: 10.3389/fpsyt.2025.1691043 (PMC12588891; doi:10.3389/fpsyt.2025.1691043)
Supplement: Supplementary file 1 [file SupplementaryFile1.docx]

**Supplementary Information**

**Table. S1.** Baseline characteristics of the study population

**Figure. S1.** The cross-lagged panel networks for T1→T2 and T2→T3 (with autoregressive edges).

**Figure. S2.** Stability of out-EI, in-EI and bridge-EI indices using the case-dropping bootstrap method for the T1 → T2 network.

**Figure. S3.** Stability of out-EI, in-EI and bridge-EI indices using the case-dropping bootstrap method for the T2 → T3 network.

**Figure. S4.** Bootstrapped 95% confidence intervals around each edge weight for the T1→T2 cross-lagged panel networks.

**Figure. S5.** Bootstrapped 95% confidence intervals around each edge weight for the T2→T3 cross-lagged panel networks.

**Figure. S6.** Center difference between out-EI for each node in T1→T2 period.

**Figure. S7.** Center difference between out-EI for each node in T2→T3 period.

**Figure. S8.** Center difference between in-EI for each node in T1→T2 period.

**Figure. S9.** Center difference between in-EI for each node in T2→T3 period.

**Figure. S10.** Center difference between bridge-EI for each node in T1→T2 period.

**Figure. S11.** Center difference between bridge-EI for each node in T2→T3 period.

**Figure. S12.** Edge weight difference tests for the T1→T2 cross-lagged panel networks.

**Figure. S13.** Edge weight difference tests for the T2→T3 cross-lagged panel networks.

| **Variable** | **N(%)/M(SD)** |
| --- | --- |
| **age** | 21.16（1.51） |
| **Residence** |  |
| Urban areas | 301(30.5%) |
| Rural areas | 687(69.5%) |
| **LBE** |  |
| with LBE | 235(23.8%) |
| without LBE | 753(76.2%) |

**Table. S1.** Baseline characteristics of the study population


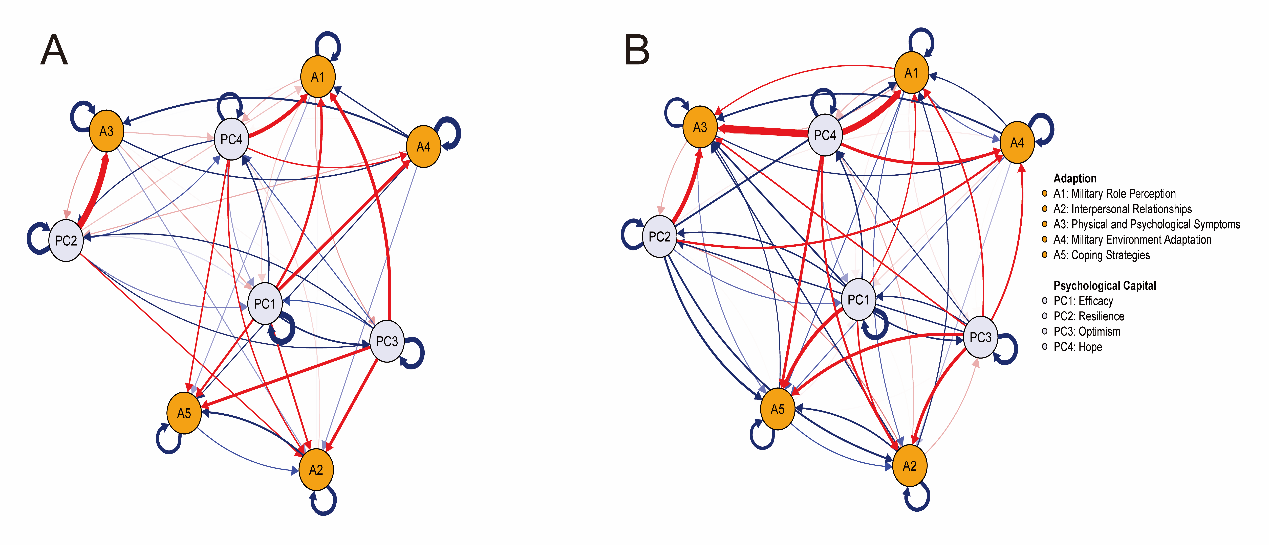


**Figure. S1.** The cross-lagged panel networks for T1→T2 and T2→T3 (with autoregressive edges).


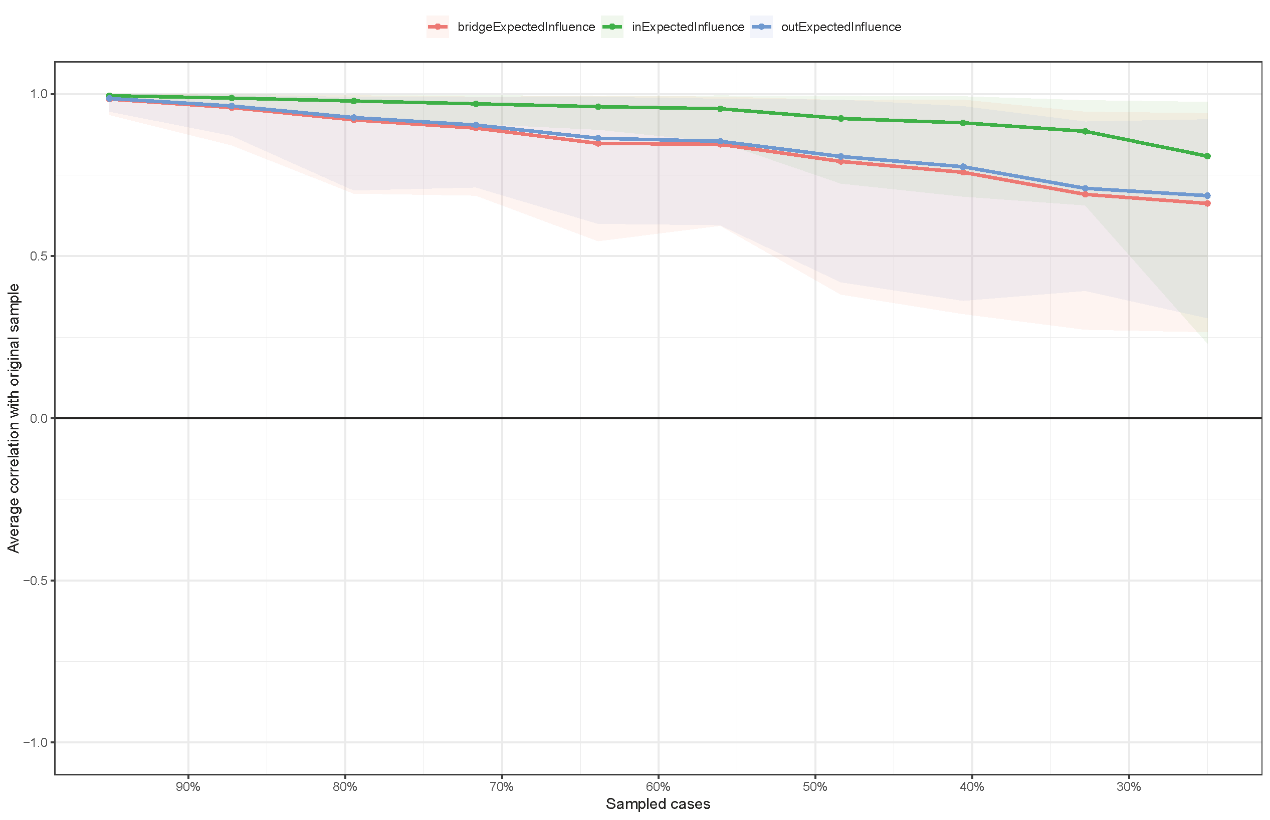


**Figure. S2.** Stability of out-EI, in-EI and bridge-EI indices using the case-dropping bootstrap method for the T1 → T2 network.


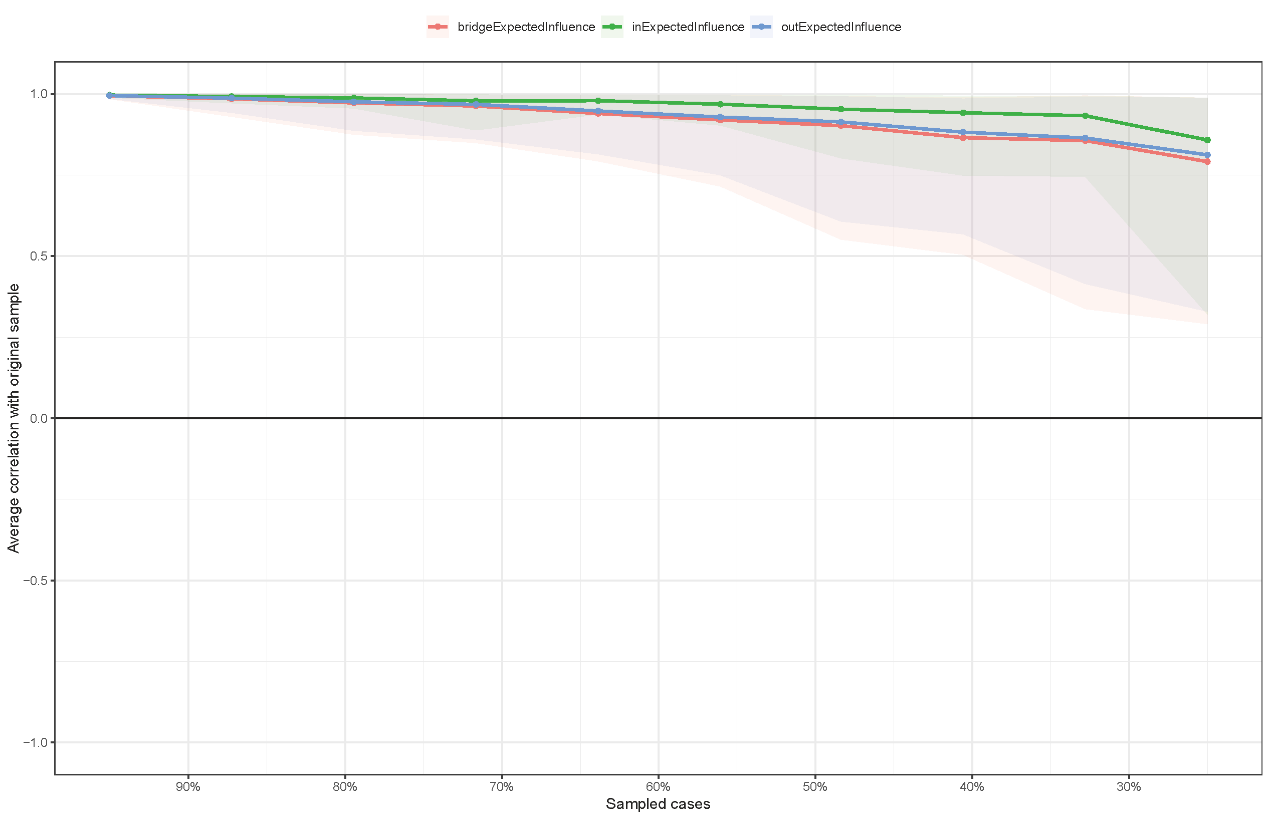


**Figure. S3.** Stability of out-EI, in-EI and bridge-EI indices using the case-dropping bootstrap method for the T2 → T3 network.


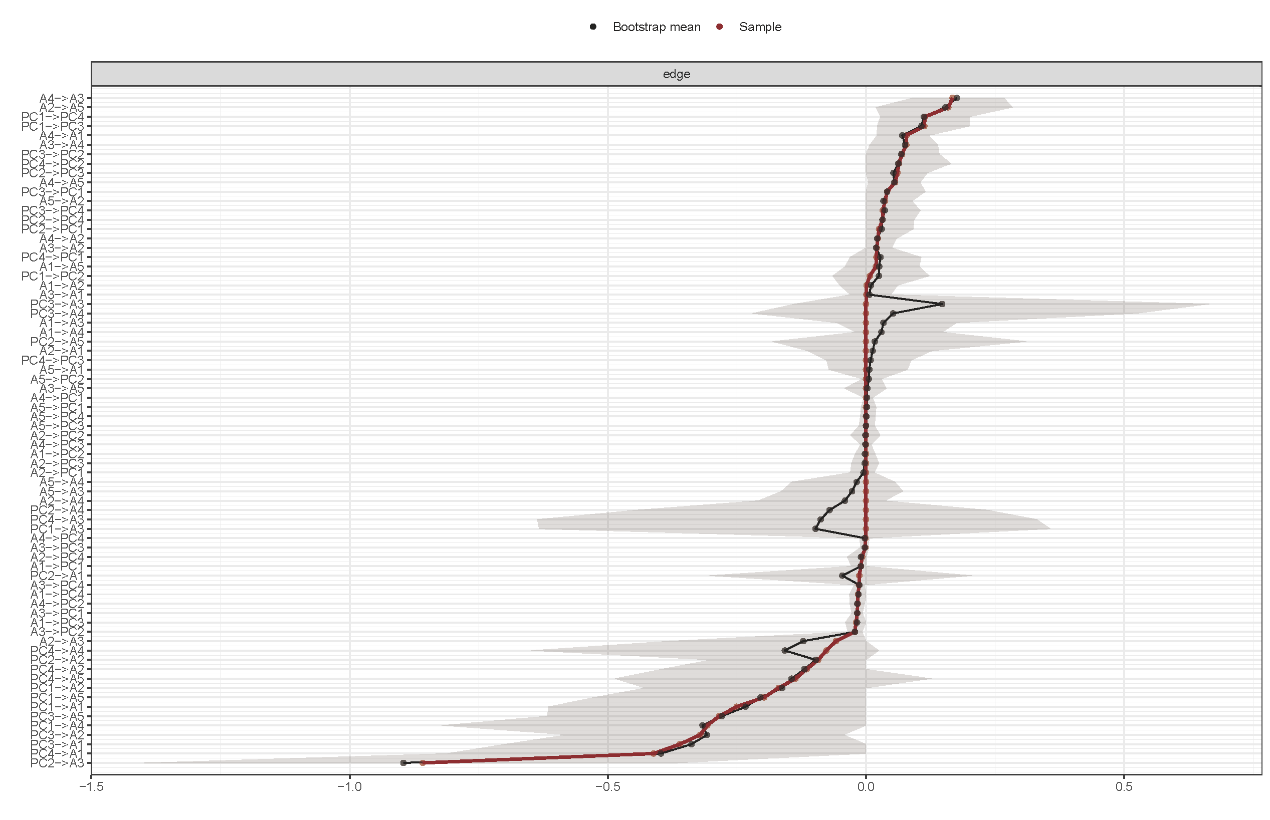


**Figure. S4.** Bootstrapped 95% confidence intervals around each edge weight for the T1→T2 cross-lagged panel networks.


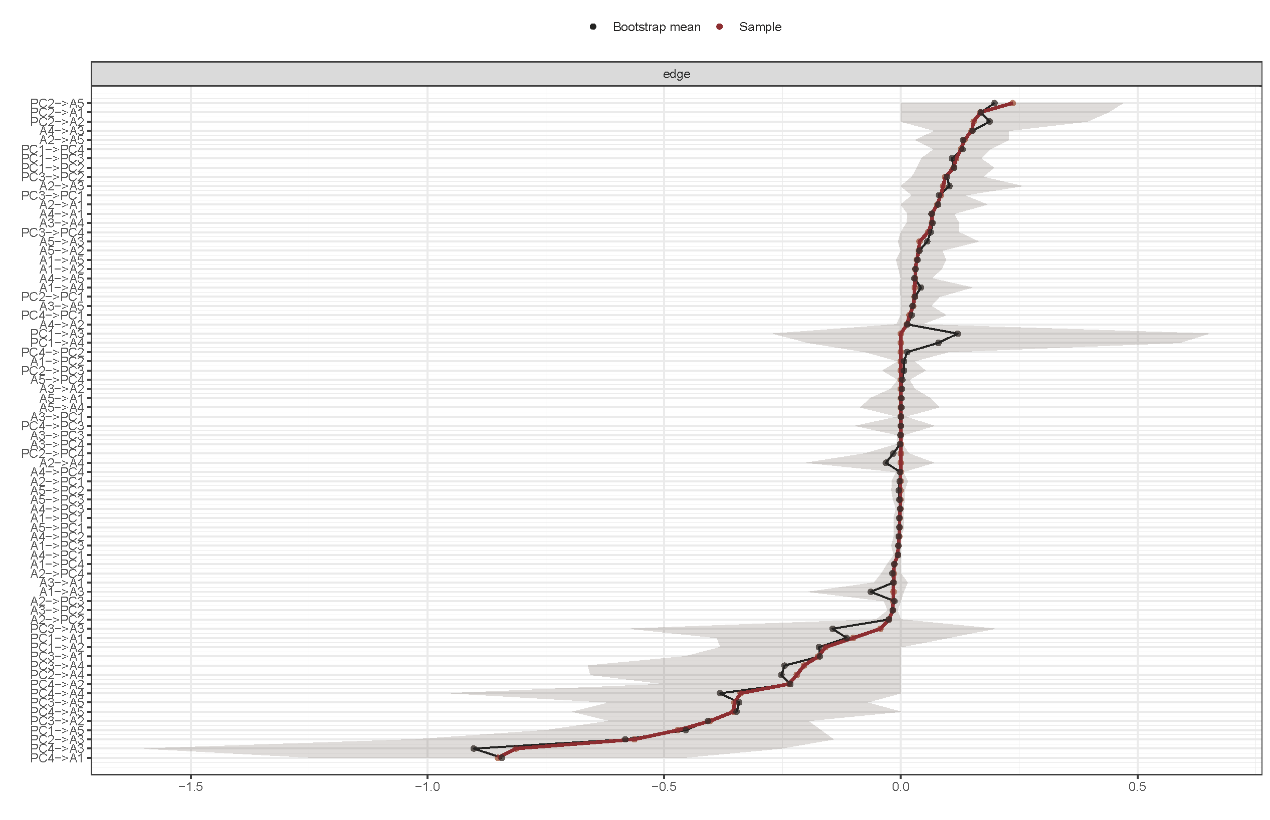


**Figure. S5.** Bootstrapped 95% confidence intervals around each edge weight for the T2→T3 cross-lagged panel networks.





**Figure. S6.** Center difference between out-EI for each node in T1→T2 period.





**Figure. S7.** Center difference between out-EI for each node in T2→T3 period.





**Figure. S8.** Center difference between in-EI for each node in T1→T2 period.





**Figure. S9.** Center difference between in-EI for each node in T2→T3 period.





**Figure. S10.** Center difference between bridge-EI for each node in T1→T2 period.





**Figure. S11.** Center difference between bridge-EI for each node in T2→T3 period.


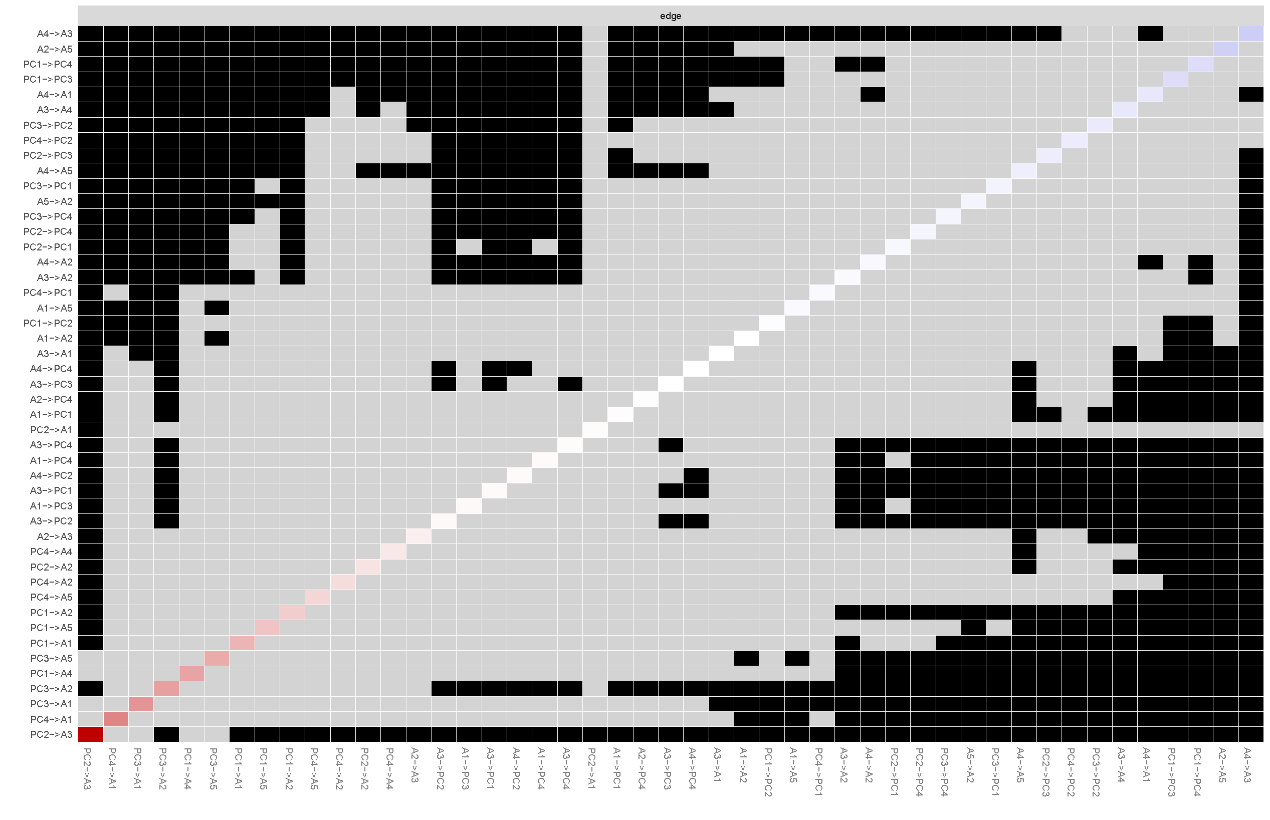


**Figure. S12.** Edge weight difference tests for the T1→T2 cross-lagged panel networks.


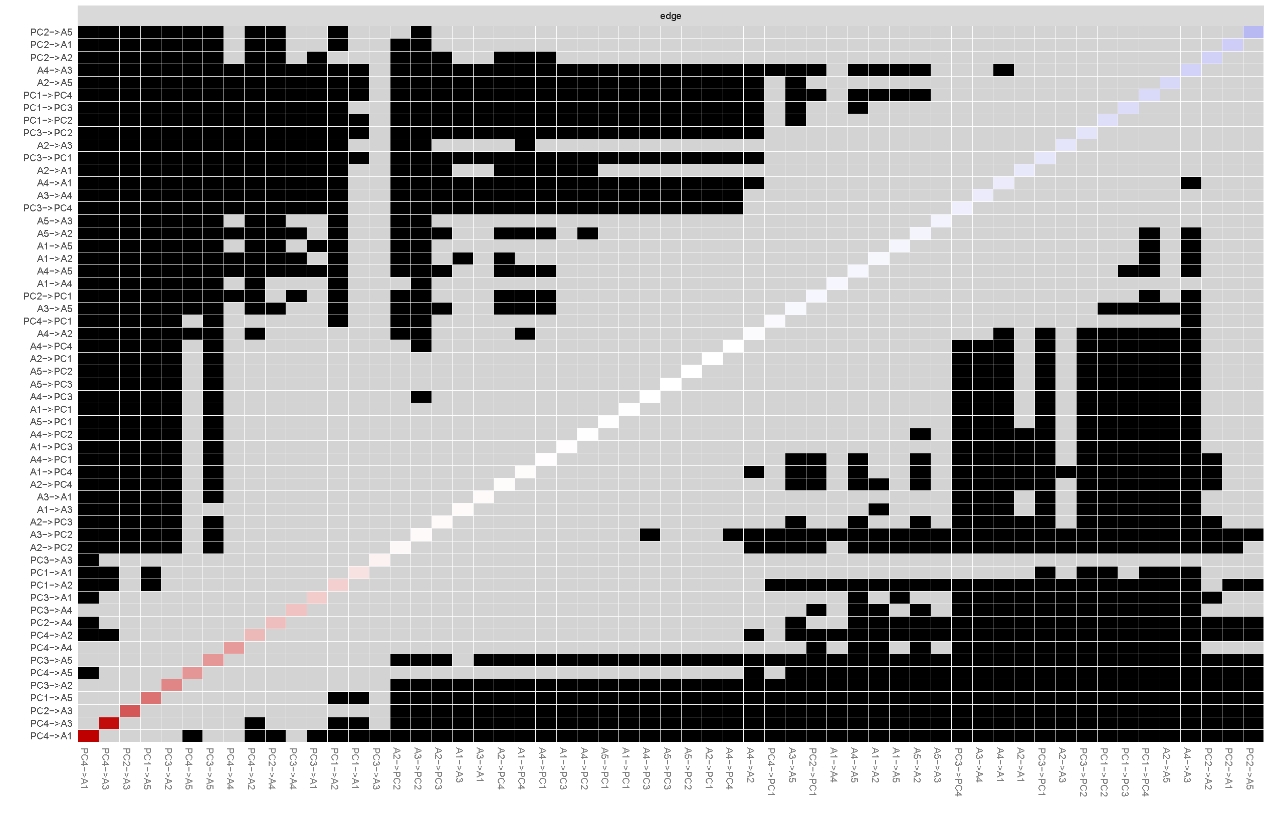


**Figure. S13.** Edge weight difference tests for the T2→T3 cross-lagged panel networks.
